# Validation of a multiplex amplification system of 19 autosomal STRs and 27 Y-STRs

**DOI:** 10.1080/20961790.2019.1665158

**Published:** 2019-11-05

**Authors:** Feng Liu, Fei Jia, Fang Sun, Bin Zhao, Hongying Shen

**Affiliations:** Liaoning Provincial Public Security Department, Shenyang, China

**Keywords:** Forensic sciences, forensic genetics, autosomal STR, Y-STR, multiplex amplification, validation

## Abstract

This article describes a newly devised autosomal short tandem repeat (STR) multiplex polymerase chain reaction (PCR) system for 19 autosomal loci (D12S391, D13S317, D16S539, D18S51, D19S433, D2S1338, D21S11, D3S1358, D5S818, D6S1043, D7S820, D8S1179, CSF1PO, FGA, TH01, TPOX, vWA, Penta D and Penta E), 27 Y-chromosome STR loci (DYS19, DYS385, DYS389I, DYS389II, DYS390, DYS391, DYS392, DYS393, DYS437, DYS438, DYS439, DYS448, DYS449, DYS456, DYS458, DYS460, DYS481, DYS518, DYS533, DYS570, DYS576, DYS635, DYS627, YGATAH4 and DYF387S1) and amelogenin with six-colour fluorescent labelling. Various parameters were evaluated, such as its accuracy, sensitivity, specificity, stability, ability to analysis of mixtures and effects of changes in the PCR-based procedures. All of the 47 selected STR loci were accurately and robustly amplified from 282 bloodstain samples. The species-specificity was high and some ability to inhibit Hematin was identified. The lowest detectable DNA amount was ≥0.125 ng. All of the male loci of the secondary component were revealed precisely when the control DNA was mixed at male/female and male/male ratios of 1:4 or more. We conclude that the present 19-plex autosomal STR and 27 Y-STR assay is both accurate and sensitive. It constitutes an additional powerful tool for forensic applications.

## Introduction

Over the course of 2 decades, autosomal STR detection has developed into a routine procedure in forensic science and plays an irreplaceable role in individual identification. Y-STRs are unique in the male genome, and the fact that paternal inheritance remains unchanged among generations. Forensic experts frequently make use of the Y chromosome’s polymorphism for identification and male population research [1]. Detection of two genetic markers, autosomal STR and Y-chromosome STR, in a single amplification can improve the test throughput, reducing the time and cost associated with testing. This system also provides a new and efficient technical method for establishing DNA and Y databases, and for performing DNA testing in actual cases. In this work, we established a novel six-dye multiplex system of 19 autosomal loci and 27 Y-chromosome loci plus the sex locus amelogenin for gender determination, and then evaluated its abilities and efficiency.

## Materials and methods

### Sample collection

A total of 282 bloodstain samples from unrelated male volunteers were collected from 2011 to 2017 after written informed consent had been obtained, and were used for concordance and accuracy studies. The positive control DNA AmpFℓSTR^®^ 9948, 9947A (ThermoFisher Scientific, Waltham, MA, USA) and PowerPlex^®^ 2800M (Promega, Madison, WI, USA) were applied to sensitivity, stability and PCR-based procedure studies. Nonhuman samples were used for analysing the species-specificity, including three male primate samples (1 ng of chimpanzee, baboon and macaque DNA) and eight nonprimate mammal samples (10 ng of cow, sheep, pig, dog, rabbit, chicken, duck and fish DNA) donated by the Laboratory Animal Centre of China Medical University (Shenyang, China).

### Locus selection and primer labelling

Our team developed multiplex amplification systems of 19 autosomal STR loci [2,3] and 20 Y-STR loci [4] in early research. Building on these two systems, we completely retained the autosomal STR loci, deleted the Y-STR loci DYS388 and DYS447, and added the loci DYS449, DYS481, DYS518, DYS533, DYS570, DYS576, DYS627 and DYF387S1 to the new system. This new system included 19 autosomal core loci, 20 Y core loci and seven Y preferred loci, conforming with the guidelines of the Ministry of Public Security, China, as well as a sex locus. The multiplex amplification system used labelling with six fluorescent dyes, FAM, HEX, TAMRA, ROX, VIC and OSD, as internal size standard labels (Table 1).

**Table 1.** Information on the 47 loci used in this study.

Locus	GenBank accession	Chromosome location	Allele range	PCR product size (bp)	Dye label
DYS392	AC011745	Yq11.223	7–21	91–133	FAM
DYS389I	AC004617	Yq11.221	9–17	142–174	
DYS456	AC010106	Yp11.2	10–23	191–243	
DYS389II	AC004617	Yq11.221	23–35	252–300	
DYS19	AC017019	Yp11.2	9–21	312–360	
DYS460^a^	AC009235	Yq11.222	6–14	367–399	
DYS518^a^	NC000024	Yq11.221	31–44	410–462	
D7S820	AC004848	7q21.11	5–16	486–530	
TH01	D00269	11p15.5	3–11.3	535–570	
D6S1043	G08539	6q15	8–24	588–652	
DYS437	AC002992	Yq11.21	10–18	85–117	HEX
DYS576	AC010104	Yp11.2	10–25	127–187	
DYS439	AC002992	Yq11.221	6–17	207–251	
YGATAH4	AC011751	Yq11.221	7–18	262–306	
DYS533	AC053516	Yq11.221	7–17	312–352	
DYS391	AC011302	Yq11.21	5–17	360–408	
DYS627^a^	NC000024	Yp11.2	14–26	429–477	
TPOX	M68651	2p25.3	5–15	485–525	
Penta E	AC027004	15q26.2	5–26	532–637	
Amel	NC000023	Xp22.2	X,Y	75,78	TAMRA
	NC000024	Yp11.2			
DYS570^a^	AC012068	Yp11.2	9–26	88–156	
DYS635	AC004772	Yq11.21	16–28	175–223	
DYS481	NC000024	Yp11.2	15–33	239–293	
DYS393	AC006152	Yp11.2	7–18	302–346	
DYS390	AC011289	Yq11.221	15–29	348–404	
DYS448	AC025227	Yq11.223	15–23	433–481	
D16S539	AC024591.3	16q24.1	5–16	487–531	
CSF1PO	X14720	5q33.1	5–16	537–581	
D19S433	G08036	19q12	8–18.2	608–650	
DYS438	AC002531	Yq11.221	6–15	86–131	ROX
DYS458	AC010902	Yp11.2	11–24	143–195	
DYF387S1^a,^^b^	NC000024	Yq11.223	32–42	213–253	
DYS385^b^	AC022486	Yq11.222	6–26	288–368	
DYS449^a^	AC051663	Yp11.2	22–42	371–451	
FGA	M64982	4q28	13–32.2	464–542	
Penta D	AP001752	21q22.3	4–17	555–620	
D8S1179	AF216671	8q24.13	7–19	82–130	VIC
D18S51	X91254	18q21.33	7–28	133–217	
D3S1358	AC099539	3p21.31	10–20	245–285	
vWA	M25858	12p13.31	11–23	291–339	
D5S818	AC008512	5q23.2	6–17	348–392	
D13S317	G09017	13q31.1	5–15	407–447	
D21S11	AP000433	21q21.1	25–38	459–511	
D2S1338	G08202	2q35	12–28	537–601	
D12S391	G08921	12p13.2	15–27	606–654	

^a^Y preferred loci; the other 20 Y-STRs were core loci.

^b^Because of palindrome structure, the application of standard PCR protocols for DYS387S1 and DYS385 results in the simultaneous amplificaiton of both copies, DYS385a and DYS385b, DYS387S1a and DYS387S1b. So both DYS385 and DYS387S1 multilocus markers are treated two separate loci.

### PCR amplification

The PCR reaction volume was 10 µL, containing 2 µL of the PCR reaction mixture, 2 µL of the primer set and 6 µL of the template DNA and water. Thermal cycling was performed with GeneAmp^®^ 9700 (ThermoFisher Scientific) using the following conditions: denaturation for 2 min at 95 °C; amplification for 30 cycles of 5 s at 94 °C, 45 s at 60 °C and 45 s at 72 °C; extension for 15 min at 60 °C; and final elongation at 4 °C forever.

### Capillary electrophoresis and data analysis

The ABI-3730XL Genetic Analyzer™ (ThermoFisher Scientific) was used to process the data with Dye Set J6 from the six dyes (FAM (blue), HEX (green), TAMRA (yellow), Rox (red), VIC (purple) and OSD (orange)) after an appropriate matrix had been created and optimised. One microliter of PCR products or allelic ladders were diluted in a mixture of 8.5 µL of Hi-Di™ Formamide (ThermoFisher Scientific) and 0.5 µL of OSD700 internal size standard. Samples were injected at 3 kV for 10 s, and separated on an ABI-3730XL Genetic Analyzer™ using POP^TM^-7 polymer (ThermoFisher Scientific) and 50-cm capillary (ThermoFisher Scientific). The bins and panels for the multiplex system were programmed for genotyping. Initial fragment sizing and allele calling were performed using GeneMappler^®^ IDX (ThermoFisher Scientific) with the peak amplitude threshold set at 50 relative fluorescence units (RFU) for all colours.

## Results and discussion

### Accuracy study

**Figure 1.**
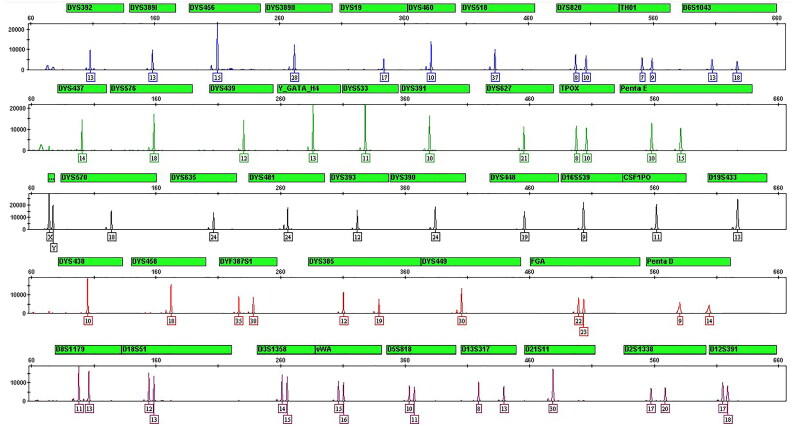
Amplification of a bloodstain sample with direct amplification.

**Figure 2.**
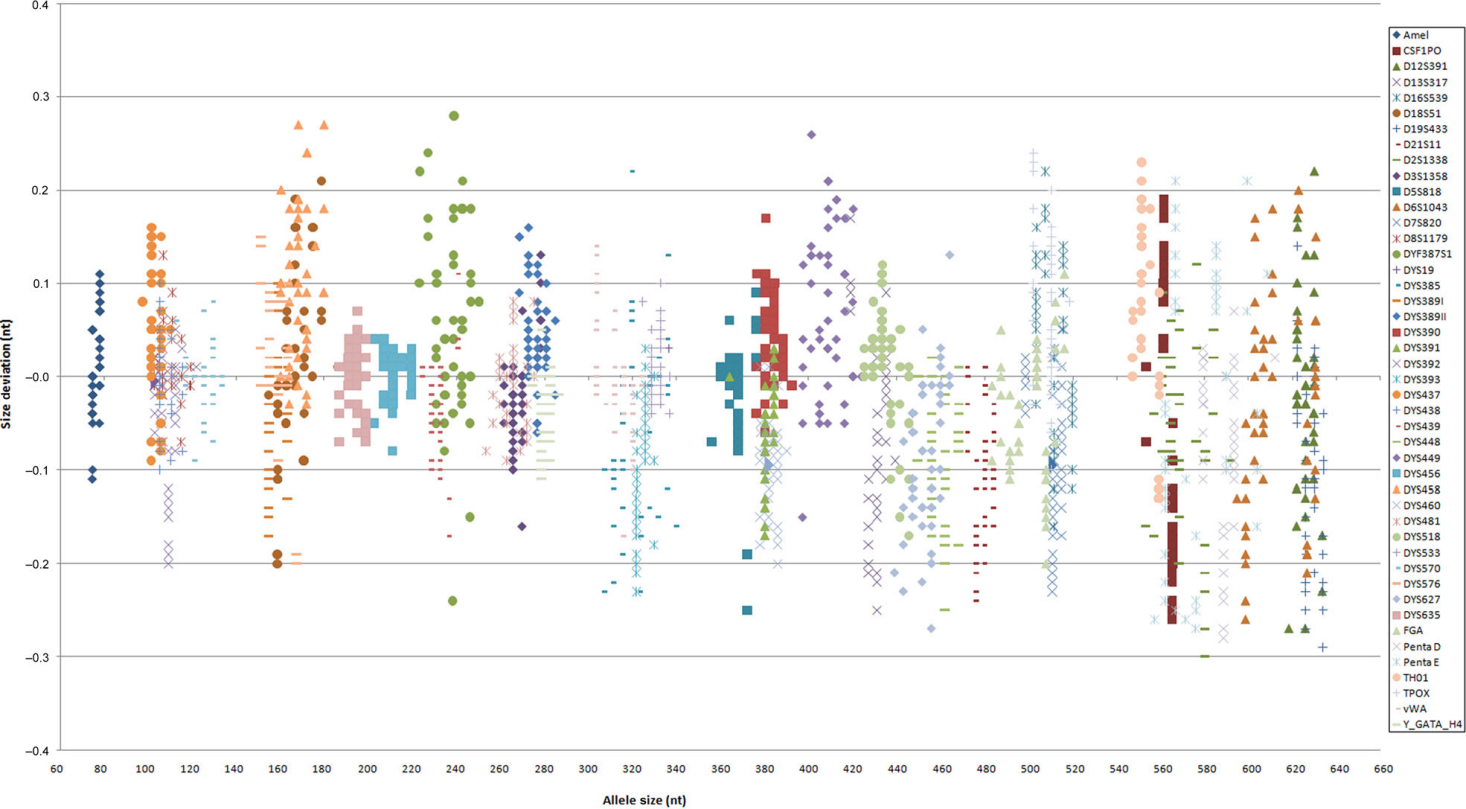
Size deviation of 2 250 alleles. The *x*-axis indicates the allele size (bp) and the *y*-axis indicates size deviation (nt). The differences were within ±0.4 nucleotides (nt), and mostly within ±0.2 nt.

**Figure 3.**
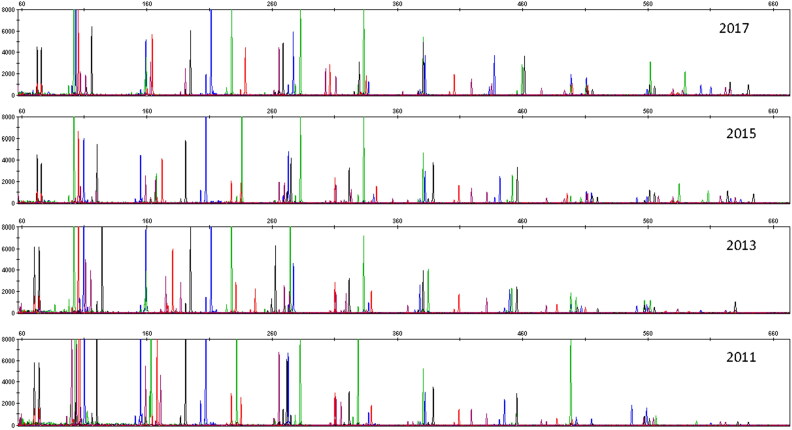
Representative electropherogram of bloodstain samples at different times. Four collection times were 2017, 2015, 2013 and 2011 (from top to bottom, respectively).

A total of 282 bloodstain samples of 1.2 mm per punch were directly amplified. Complete profiles were obtained from all samples (Figure 1). The accuracy of this multiplex amplification system was evaluated by calculating the size differences between sample alleles and allelic ladder alleles. The differences were within ±0.4 nucleotides (nt), and mostly within ±0.2 nt (Figure 2). By combining STR loci in the GlobalFiler™ kit (ThermoFisher Scientific), PowerPlex^®^ Fusion 6C kit (Promega) and the Y Filer™ Plus kit (ThermoFisher Scientific), a total of 47 loci can be obtained in our multiplex system. Upon comparison, the STR genotypes from 282 samples obtained using the multiplex system were in accordance with those from the three kits aforementioned. This demonstrates that our multiplex system has good accuracy. The findings also showed that the peak height of larger loci decreased as storage time increased (Figure 3).

### Sensitivity study

**Figure 4.**
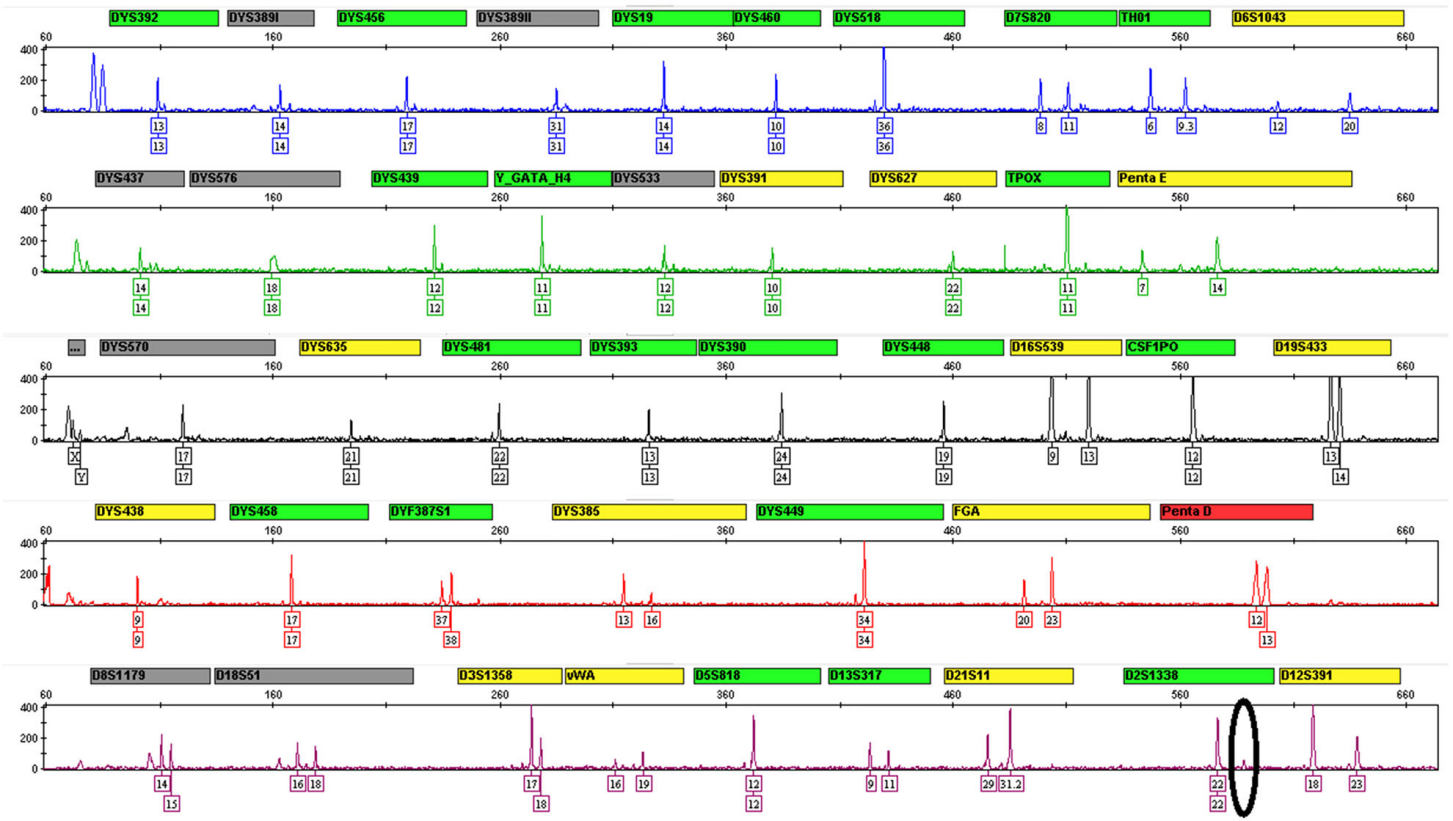
Amplification of 62.5 pg of 2800M DNA control sample. The black ellipse showed that D2S1338 locus dropped out in one incomplete profile.

The positive control 2800M DNA was used as a sample for testing the sensitivity. After quantification, studies were performed in duplicate using final DNA amounts ranging from 31.25 pg to 8 ng. The results showed that the optimal quantity of DNA for this system ranged from 125 pg to 4 ng, with average peak heights ranging from 826 to 7 034 RFU. As the amount of DNA decreased to 62.5 pg, the D2S1338 locus dropped out in one incomplete profile and the average peak height was 283 RFU (Figure 4). Upon reducing the quantity to 31.25 pg, the average percentage profile was 69.53% with average peak height of 125 RFU. Conversely, when the DNA amount was increased to 8 ng, complete profiles were achieved with average peak height of 8 004 RFU (peak heights of some loci above 25 000 RFU) along with the appearance of bleed-through at homozygous loci, which were likely to lead to inaccurate results. The detection limit of the multiplex is 125 pg, which is suitable for genotyping of trace sample evidence.

### Specificity study

**Figure 5.**
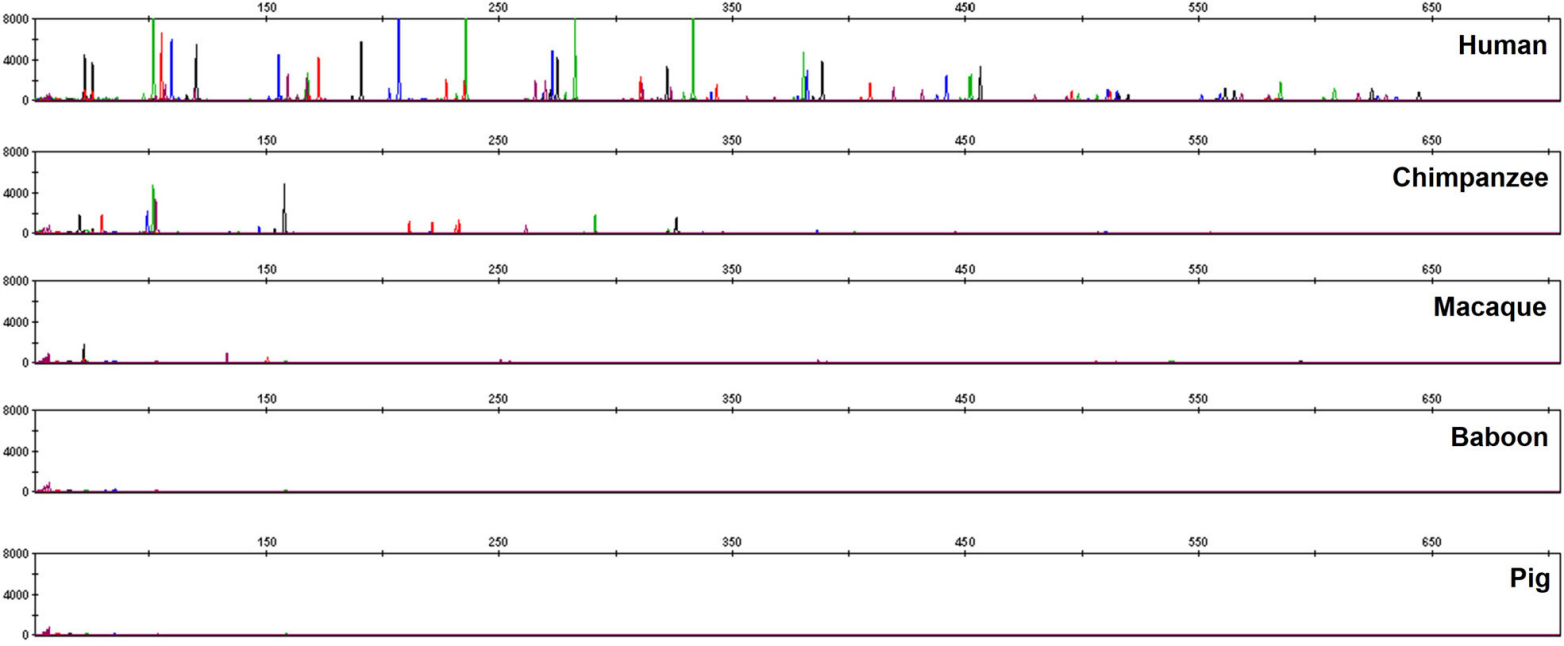
Representative electropherogram of species-specificity study. Three kinds of DNA were showed, including human DNA, nonhuman primates (chimpazee, macaque and baboon) and nonprimate mammal pig from top to bottom, respectively.

To ensure that the multiplex system only amplifies human DNA, we assessed its potential to detect DNA from 11 nonhuman animal species in duplicate, including three nonhuman male primates (1 ng each from chimpanzees, baboon and macaque) and eight male nonprimate mammals (10 ng each from cow, sheep, pig, dog, rabbit, chicken, duck and fish). The results showed that no genetic profiles were obtained for the DNA from the eight nonprimate mammals. The profiles of the three nonhuman primate DNA samples revealed fragments. Among them, the chimpanzee STRs with the highest homology to human STRs showed the most nonspecific amplification. A total of 36 peaks were assigned at 28 loci, of which 52.8% (19/36) were off-ladder peaks, so the profiles could be distinguished from human; macaque showed 20 nonspecific amplification and 40% off-ladder peaks; at least, baboon yielded the only five peaks. Therefore, this system is suitable for human identification with good species-specificity, and is not affected by forensically relevant nonhuman DNA contamination (Figure 5).

### Mixture study

After quantification of the positive control 9948 and 2800M DNA, male/male mixtures of the control DNA were examined in duplicate at various ratios (9:1, 4:1, 1:1, 1:4 and 1:9), while keeping the total amount of mixed DNA input constant at 1 ng. As the ratios increased, there was a decrease in the proportion of minor alleles that could be identified. The minor component (200 pg) at 4:1 and 1:4 ratios was completely identified. At ratios of 9:1 and 1:9, the minor component (100 pg) yielded 29% (11/38) and 44% (15/34) partial profiles, respectively.

**Figure 6.**
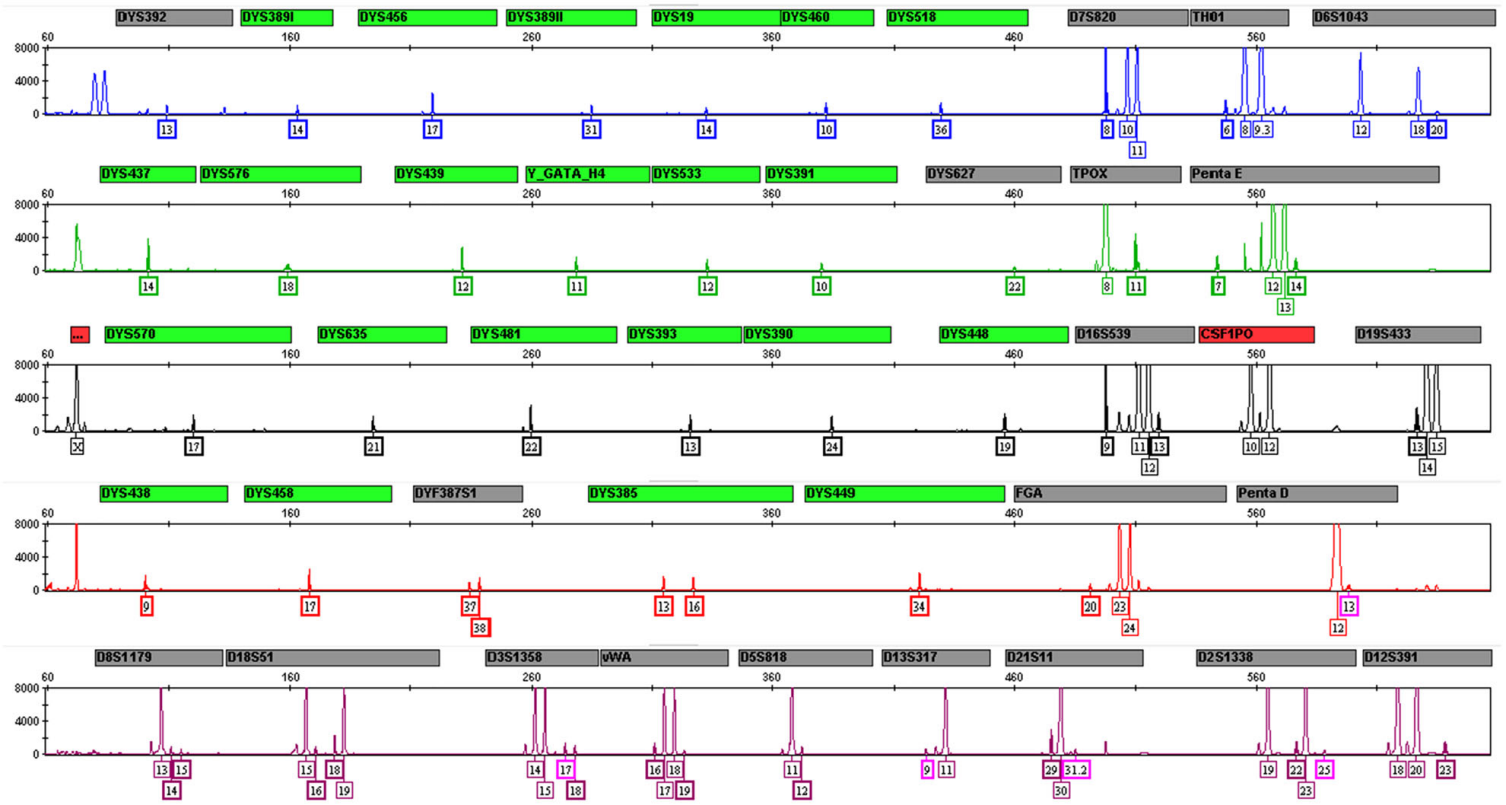
Profile of a male/female mixed sample of 2800M/9947A DNA (1:4). All of autosomal and Y alleles of the male component could be typed when the male/female ratio was 1:4. Bold peaks showed nonoverlapping alleles of the male 2800M DNA from mixture amplification.

After the male 2800M DNA and female 9947A DNA had been quantified, male/female mixtures were examined in duplicate at various ratios (1:1, 1:2, 1:4, 1:9, and 1:19), while keeping the total amount of mixed DNA input constant at 1 ng. When the ratio was no less than 1:4, all of the autosomal and Y alleles of the minor component could be typed (Figure 6). At the ratio of 1:9, all Y alleles of the minor component could be detected, but 52% (13/25) partial profiles of minor autosomal alleles could be detected. At the ratio of 1:19, no autosomal alleles of the male DNA were detected, but 74% (20/27) partial profiles of minor Y loci were identified.

Our multiplex system has clear advantages in the characterization of DNA mixtures, and is more reliable for analysing the male component in such mixtures. This enables inferences to be made about the number of male individual genotypes and determination of the presence of male components in mixtures containing material from a large number of females.

### Stability study

Hematin has been identified as a PCR inhibitor in DNA samples extracted from bloodstains. As the multiplex system is used mainly for direct amplification in database construction, hematin was tested to evaluate the tolerance of the multiplex to Hematin inhibition. Porcine hematin (Sigma-Aldrich, St. Louis, MO, USA) was diluted to 25 mg/mL in 1 mol/L NaOH and then supplemented with 1 ng of 2800M DNA prior to PCR at concentrations varying from 0 to 100 µmol/L in duplicate. The results showed that the average peak height of larger alleles decreased at 50 µmol/L hematin, although complete profiles were obtained. When the concentration of hematin increased to 100 µmol/L, no profile was obtained.

### PCR-based procedures

#### Reaction volume

Reaction volumes of 5, 10 and 25 µL were tested in duplicate using 1 ng of control 2800M DNA. The full profiles were obtained across all volumes. As the volume increased, the peak heights decreased from 3 997, 3 073 to 1 022 RFU, and the balance was enhanced. These results are in accordance with previous studies [2]. When the template is a low-copy-number (LCN) sample, the signal increases and profiles are more balanced with a smaller final reaction volume.

#### Annealing temperature

An optimised annealing temperature of 58 °C was determined using primer sequences, but the temperatures of different PCR thermal cyclers may differ slightly. To determine the appropriate annealing temperature, annealing was conducted here in the range from 54 °C (−4 °C) to 62 °C (+4 °C) using 1 ng of control 2800M DNA in duplicate to ensure the stability and accuracy of genotyping in the multiplex system. The results revealed no change in the precision and accuracy, no nonspecific amplification and no allele dropout under these annealing temperature conditions, but the amplification balance was affected by the annealing temperature. Compared with that at the other four annealing temperatures, the balance was better at 58 °C.

#### Cycle number

The optimal cycle number of the multiplex is 30. Amplifications of 1 ng of control 2800M DNA were performed in duplicate with a range of cycle numbers from 26 (−4) to 34 (+4). Under the conditions tested, full profiles were reliably generated across all cycle numbers, and there was no nonspecific amplification or allele dropout. The signal intensity increased with increasing cycle number, from 480 (26 cycles), 813 (28 cycles), 4 608 (30 cycles), 8 669 (32 cycles) to 10 607 RFU (34 cycles). However, it is recommended to increase the number of cycles appropriately, such as to 32, when the input amount is below the detection threshold, in order to obtain an optimal multiplex profile.

#### Primer set concentration

Amplifications of 1 ng of control 2800M DNA were tested in duplicate with a series of primer set concentrations (0.5×, 1.0×, 2.0×). Full profiles were obtained, with neither nonspecific amplification nor allele dropout occurring at the different concentrations tested. The average peak heights increased linearly from 490 to 7 040 RFU with increasing concentration. Therefore, the optimal primer amount is 1×, in consideration of the detection ability and cost-efficiency.

## Conclusion

In routine DNA typing methods, autosomal STRs and Y-STRs are usually detected separately during the inspection of forensic evidence. The Y-STR loci have been added to some autosomal STR kits [5,6], such as GlobalFiler™ kit [7] and PowerPlex^®^ Fusion 6C kit [8], which are currently widely used in laboratories. Owing to the limited number of Y loci, the use of Y-chromosome-specific alleles can only avoid the misjudgement of gender caused by allelic mutations of amelogenin-Y. Adding more Y-STR loci in a single amplification has thus become a particular focus of research on the establishment of multiplex amplification systems [9,10].

A new method of detecting autosomal and Y loci was developed in this study. It combines 19 autosomal STRs, 27 Y-STRs and amelogenin, and can simultaneously obtain both autosomal STR and Y-STR genotyping information through a single amplification. Our multiplex system includes the highest number of autosomal STRs and Y-STRs among the systems established to date. The Y-STR information can be used to rapidly screen suspects, after which the autosomal STR information can confirm the suspects, improving the time-efficiency of the investigation. In terms of database construction, this approach also satisfies the locus requirements of the DNA and Y databases constructed in China. This reduces the time and cost associated with detection, while leading to greater efficiency of database construction.

Optimal performance of the system designed here was also established in accordance with the standard GA/T815-2009 “Criterion for the human fluorescent STR multiplex PCR reagent” [11]. Complete profiles were obtained from 0.125 ng of DNA. The minor component (0.2 ng) at mixture ratios of 1:4 and 4:1 could be typed robustly. Full profiles of 1 ng of DNA in the presence of 50 µmol/L hematin were generated. In comparison with the previous 20-locus multiplex system [2], there was poor balance of peak height of the 47-locus system because of the excessive number of loci. However, the new system could still obtain complete profiles and accurate results during challenging PCR procedures.

In another study [12], the multiplex system was evaluated in forensic applications. The system has been proven to applicable for both direct amplification of bloodstain samples and detection of evidence samples from criminal cases. And the 46 STR loci have been proved to highly genetically polymorphic in the Han population in Liaoning Province, and effectively valuable for discriminating individuals and testing kinship.

In summary, a fluorescent multiplex amplification system based on 47 DNA loci was successfully determined. This system includes 19 autosomal STRs, 27 Y-STRs and amelogenin and is a highly informative, powerful and effective tool for forensic applications. It should become an ideal tool for forensic DNA typing, database construction and paternity testing.
